# Effect of empagliflozin on total myocardial infarction events by type and additional coronary outcomes: insights from the randomized EMPA-REG OUTCOME trial

**DOI:** 10.1186/s12933-024-02328-6

**Published:** 2024-07-11

**Authors:** David Fitchett, Bernard Zinman, Silvio E. Inzucchi, Christoph Wanner, Stefan D. Anker, Stuart Pocock, Michaela Mattheus, Ola Vedin, Søren S. Lund

**Affiliations:** 1 Division of Cardiology, St Michael’s Hospital, University of Toronto, Toronto, ON Canada; 2 Lunenfeld-Tanenbaum Research Institute, Mount Sinai Hospital, University of Toronto, Toronto, ON Canada; 3 Section of Endocrinology, Yale University School of Medicine, New Haven, CT USA; 4 Würzburg University Clinic, Würzburg, Germany; 5 Department of Cardiology (CVK) and Berlin Institute of Health Center for Regenerative Therapies (BCRT), German Centre for Cardiovascular Research (DZHK) Partner Site Berlin, Charité Universitätsmedizin Berlin, Berlin, Germany; 6 Institute of Heart Diseases, Wrocław Medical University, Wrocław, Poland; 7 Department of Medical Statistics, London School of Hygiene & Tropical Medicine, London, UK; 8 Boehringer Ingelheim Pharma GmbH & Co. KG, Ingelheim, Germany; 9 Boehringer Ingelheim AB, Stockholm, Sweden; 10 Boehringer Ingelheim International GmbH, Ingelheim, Germany

**Keywords:** Diabetes mellitus, Type 2, Myocardial infarction, Sodium-glucose transporter 2 inhibitors

## Abstract

**Background:**

The effect of empagliflozin, a sodium-glucose-co-transporter-2 inhibitor, on risk for myocardial infarction has not been fully characterized.

**Methods:**

This study comprised prespecified and post-hoc analyses of the EMPA-REG OUTCOME trial in which 7020 people with type 2 diabetes (T2D) and cardiovascular disease [mostly atherosclerotic (ASCVD)] were randomized to empagliflozin or placebo and followed for a median 3.1 years. We assessed the effect of empagliflozin on total (first plus recurrent) events of centrally adjudicated fatal and non-fatal myocardial infarction (MI) using a negative binomial model with robust confidence intervals (CI) that preserves randomization and accounts for the within-patient correlation of multiple events. Post hoc, we analyzed types of MI: type 1 (related to plaque-rupture/thrombus), type 2 (myocardial supply–demand imbalance), type 3 (sudden-death related, i.e. fatal MI), type 4 (percutaneous coronary intervention-related), and type 5 (coronary artery bypass graft-related). MIs could be assigned to > 1 type.

**Results:**

There were 421 total MIs (including recurrent); 299, 86, 26, 19, and 1 were classified as type 1, 2, 3, 4, and 5 events, respectively. Overall, empagliflozin reduced the risk of total MI events by 21% [rate ratio for empagliflozin vs. placebo, 0.79 (95% CI, 0.620–0.998), *P* = 0.0486], largely driven by its effect on type 1 [rate ratio, 0.79 (95% CI, 0.61–1.04)] and type 2 MIs [rate ratio, 0.67 (95% CI, 0.41–1.10)].

**Conclusions:**

In T2D patients with ASCVD, empagliflozin reduced the risk of MIs, with consistent effects across the two most common etiologies, i.e. type 1 and 2.

Trail Registration: URL: https://www.clinicaltrials.gov; Unique identifier: NCT01131676.

**Supplementary Information:**

The online version contains supplementary material available at 10.1186/s12933-024-02328-6.

## Background

Type 2 diabetes (T2D) confers high risk for myocardial infarction (MI) and other cardiovascular (CV) events [1]. In CV outcomes trials, sodium-glucose transporter (SGLT) inhibitors such as empagliflozin, initially developed as glucose-lowering agents, reduced major adverse cardiovascular events, CV deaths, heart failure, and kidney disease outcomes in T2D patients at high CV risk [2–7], and in patients with chronic heart failure or kidney disease, including individuals who did not have T2D [8–13].

Analyses of first and total (first plus recurrent) events in CV outcomes trials showed that SGLT inhibitors, including empagliflozin, also reduce coronary events, including MI [6, 14, 15]. The most common types of MI arise from classical atherothrombosis (type 1 MI) or from imbalances in myocardial supply–demand (type 2 MI) [16]. However, the effect of empagliflozin on different types of MI is unclear.

Here, we analyze the effect of empagliflozin on total MIs by type in T2D patients with atherosclerotic CV disease in the EMPA-REG OUTCOME trial, and additional coronary outcomes.

## Methods

The EMPA-REG OUTCOME trial randomized T2D patients with established CV disease typically of atherosclerotic origin (MI, stroke, coronary artery disease, and/or peripheral artery disease) to empagliflozin 10 mg/day, 25 mg/day, or placebo [2]. The primary endpoint was time to first occurrence of major adverse cardiovascular events (composite of CV death, non-fatal MI, or non-fatal stroke). All CV and mortality outcomes were centrally adjudicated in a blinded manner by independent specialists [2].

Analyses of first and total MIs overall (fatal and non-fatal events) were prespecified [15]. Post hoc, we also analyzed a main coronary outcome (composite of MI or coronary revascularization) and an expanded coronary outcome (composite of MI, coronary revascularization, or hospitalization for unstable angina). Also post hoc, we analyzed the following types of MI [2]: type 1 (related to atherosclerotic plaque and thrombus); type 2 (related to imbalance in myocardial supply–demand); type 3 (sudden death-related, i.e. fatal MI); type 4 (percutaneous coronary intervention-related); and type 5 (coronary artery bypass graft-related). MIs could be assigned to > 1 type (e.g., type 3 [fatal] MI of type 1 etiology); however, each MI—even if assigned to > 1 type—could only be included in the individual analyses as one event but could be included as different types of MI in different analyses if assigned to > 1 type.

Empagliflozin dose groups were pooled for all analyses, which included all patients who received ≥ 1 dose of study drug (modified intention-to-treat (mITT) population). We calculated 95% confidence intervals (CIs) and *P* values without adjustment for multiplicity. The rate of total events was analyzed using negative binomial regression as prespecified [15]. The model included terms for baseline age, sex, body mass index, glycated hemoglobin, estimated glomerular filtration rate, region, and treatment group using the natural logarithm of the observation time as an offset variable. CIs were based on robust error variance estimators to account for within-participant correlation. First events were analyzed by a Poisson model with similar factors as the negative binomial model. Subgroup analyses for the outcomes showing an overall statistically significant treatment effect (total MIs, main coronary outcome, and the expanded coronary outcome) were performed according to baseline kidney function (eGFR < 60, 60 -< 90, and ≥ 90 ml/min/1.73m^2^), baseline use of glucagon-like peptide-1 (GLP-1) analogues (a glucose-lowering drug with demonstrated effects on CV outcomes) [1] and the most commonly used glucose-lowering therapies (metformin, sulfonylurea (SU), and insulin) including a factor for the respective subgroup and treatment by subgroup interaction. On-treatment sensitivity analyses (using only events that occurred up to patient’s last intake of trial medication) were performed for the outcomes showing an overall statistically significant treatment effect. To account for informative censoring because of mortality, or specifically mortality other than fatal MI caused by the effect of empagliflozin on mortality, we did sensitivity analyses using a semi-parametric joint frailty model (with treatment as a covariate) [17]. Some analyses were previously reported and are shown here for context [2, 15]. Significance was determined on the basis of an α-level of 0.05 without correction for multiple testing.

## Results

As previously reported, 7020 patients were randomized and followed for a median 3.1 years; baseline characteristics were similar between treatment groups [2, 15]. A total of 6891 patients (98.2%) used glucose-lowering medications at baseline—most commonly metformin [n = 5193 (74.0%)], SUs [n = 3006 (42.8%)], and insulin [n = 3387 (48.2%)]. A total of 196 patients (2.8%) used a GLP-1 agonist at baseline [15]. A total of 6667 patients (95.0%) used anti-hypertensive medication at baseline (most commonly ACE inhibitors/ARBs [(n = 5666 (80.7%)], beta-blockers [n = 4554 (64.9%)], diuretics [n = 3035 (43.2%)], and calcium channel blockers [n = 2317 (33.0%), and a total of 5684 (81.0%) used lipid-lowering medications at baseline (most commonly statins [n = 5403 (77.0%)]—no patient received proprotein convertase subtilisin/kexin type 9 (PCSK9) inhibitors]).

Overall, 421 MIs occurred in 349 patients: 299 type 1 MIs in 260 patients; 86 type 2 MIs in 75; 26 type 3 MIs in 26; 19 type 4 MIs in 19; and 1 type 5 MI. Among these, 10 MIs were assigned to > 1 MI type: all type 1 (atherothrombotic) and type 3 (fatal). The numbers of patients by number of MI events are shown in Table 1. Placebo patients who subsequently experienced type 1 or 2 MIs during the trial were slightly older and had a more adverse baseline CV risk profile than those who did not experience MI (Table 2).

**Table 1:** Number of myocardial infarctions

	Empagliflozin (N = 4687)	Placebo (N = 2333)
Number of myocardial infarctions		
None	4464 (95.2)	2207 (94.6)
1	187 (4.0)	103 (4.4)
2	32 (0.7)	19 (0.8)
≥ 3	4 (0.1)	4 (0.2)

Data are n (%) of patients

**Table 2:** Baseline characteristics of placebo-treated patients by MI type

	≥ 1 type 1 MI* (N = 95)	≥ 1 type 2 MI* (N = 32)	No MI (N = 2207)
Age, years	65.2 (8.3)	66.2 (8.8)	63.1 (8.8)
Male, n (%)	70 (73.7)	20 (62.5)	1590 (72.0)
Smoking status, n (%)			
Current	13 (13.7)	7 (21.9)	283 (12.8)
Ex-smoker	45 (47.4)	15 (46.9)	1015 (46.0)
Never smoked	37 (38.9)	10 (31.3)	909 (41.2)
> 10 years since T2D diagnosis, n (%)	64 (67.4)	19 (59.4)	1260 (57.1)
Body mass index, kg/m^2^	30.4 (4.9)	30.5 (5.4)	30.7 (5.3)
eGFR, mL/min/1.73 m^2^	71.5 (18.8)	61.8 (16.9)	74.1 (21.1)
LDL-C, mg/mL	89.0 (38.8)	83.8 (40.0)	84.8 (35.2)
HbA1c, %	8.0 (0.8)	7.9 (0.8)	8.1 (0.8)
SBP, mmHg	139.7 (20.8)	139.7 (22.0)	135.6 (17.0)
DBP, mmHg	75.7 (9.7)	75.1 (10.7)	76.9 (10.2)
UACR, n (%)			
30 to 300 mg/g	29 (30.5)	13 (40.6)	633 (28.7)
> 300 mg/g	15 (15.8)	3 (9.4)	241 (10.9)
Concomitant medication, n (%)			
Antihypertensives	91 (95.8)	31 (96.9)	2100 (95.2)
Lipid-lowering	81 (85.3)	28 (87.5)	1753 (79.4)
Anticoagulants	88 (92.6)	30 (93.8)	1973 (89.4)
Glucose-lowering medication, n (%)			
Monotherapy	28 (29.5)	12 (37.5)	651 (29.5)
Dual therapy	41 (43.2)	15 (46.9)	1091 (49.4)
Insulin	53 (55.8)	18 (56.3)	1066 (48.3)
Previous CV disease, n (%)			
MI	68 (71.6)	22 (68.8)	997 (45.2)
CABG	30 (31.6)	14 (43.8)	523 (23.7)
CAD^†^	89 (93.7)	28 (87.5)	1646 (74.6)
Peripheral artery disease	18 (18.9)	10 (31.3)	454 (20.6)
Stroke	16 (16.8)	5 (15.6)	531 (24.1)
Heart failure	14 (14.7)	9 (28.1)	222 (10.1)

Data are mean (SD) unless stated otherwise. SI conversion factor: to convert LDL-C to mmol/L, multiply by 0.0259

*CABG* coronary artery bypass graft, *CAD* coronary artery disease, *CV* cardiovascular, *DBP* diastolic blood pressure, *eGFR* estimated glomerular filtration rate, *HbA1c* glycated hemoglobin, *LDL-C* low-density lipoprotein cholesterol, *MI* myocardial infarction, *SBP* systolic blood pressure, *SD* standard deviation, *T2D* type 2 diabetes, *UACR* urine albumin-to-creatinine ratio

^*^Patients can be counted in more than 1 column

^†^CAD was defined as history of MI, CABG, and/or multi/single vessel CAD

**Fig. 1:**
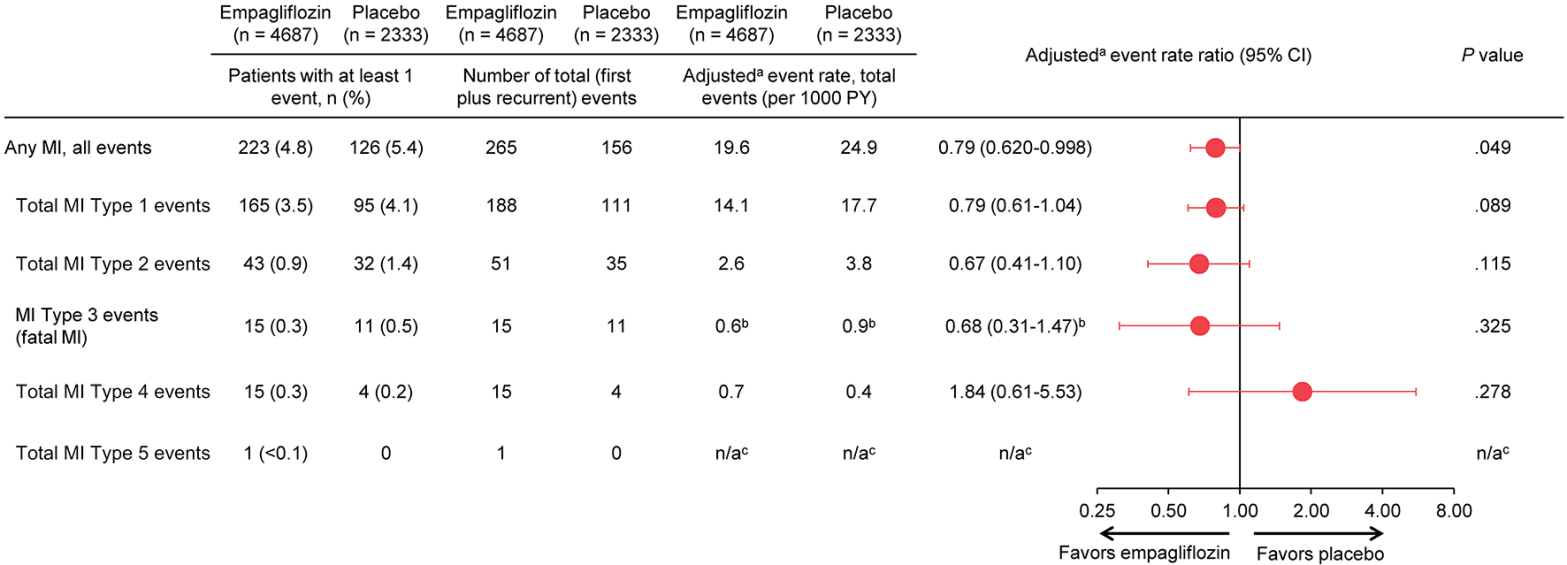
**Risk of Total MI Events by Type. **Types of MI are not mutually exclusive. *BMI* indicates body mass index, *CI* confidence interval, *eGFR* estimated glomerular filtration rate, *HbA1c* glycated hemoglobin, *MI* myocardial infarction, *n/a* not available, *PY* patient-years. ^a^Negative binomial model includes age as a linear covariate and treatment, sex, baseline BMI category, baseline HbA1c category, baseline eGFR category and geographical region as fixed effects with log (observation time) as offset. ^b^Poisson regression model includes age as a linear covariate and treatment, sex, baseline BMI category, baseline HbA1c category, baseline eGFR category and geographical region as fixed effects with log (time to event) as offset. ^c^Number of events too small to conduct analyses

Overall, empagliflozin reduced the relative risk of total MIs by 21% [rate ratio (RR) for empagliflozin versus placebo, 0.79 (95% CI, 0.620–0.998); *P* = 0.0486] (Fig. 1 and eFigure a), as previously reported [15]. The overall reduction in total MIs by empagliflozin was driven mainly by its effect on type 1 [RR, 0.79 (95% CI, 0.61–1.04)] and type 2 MIs [RR, 0.67 (95% CI, 0.41–1.10)] (Fig. 1).

Furthermore, empagliflozin elicited a 20% relative risk reduction for total events of the main coronary outcome [MI or coronary revascularization: RR, 0.80 (95% CI, 0.67–0.95)] (eFigure b) and a 17% relative risk reduction for the expanded coronary outcome [main coronary outcome or hospitalization for unstable angina: RR, 0.83 (95% CI, 0.70–0.99)] (eFigure c) [15]. Results were consistent for coronary revascularization alone [RR, 0.85 (95% CI, 0.71–1.03)], but there was no effect on hospitalization for unstable angina [RR, 1.03 (95% CI, 0.76–1.41)] (eFigure d and e, respectively) [15]. The effect on MI and the coronary outcomes was evident within ~ 3 months and sustained (eFigure) [15].

Subgroup analyses showed a consistent treatment effect of empagliflozin versus placebo for total MIs, the main coronary outcome, and the expanded coronary outcome according to baseline kidney function (eGFR < 60, 60-< 90, and ≥ 90 ml/min/1.73m^2^) (p for interaction: 0.1922, 0.4450, 0.6909, for the three outcomes, respectively), baseline use of metformin (p for interaction: 0.2463, 0.4891, 0.6626), SU (p for interaction: 0.7182, 0.1187, 0.0513), insulin (p for interaction: 0.9358, 0.5576, 0.8534), and GLP-1 analogues (p for interaction 0.3024, 0.4356, 0.3895).

On-treatment sensitivity analyses showed a consistent treatment effect for total MIs [RR 0.85 (95% CI, 0.65–1.10)], p = 0.2087, total of 316 events (75.1% of events in the mITT analysis), the main coronary outcome [RR 0.80 (95% CI, 0.66–0.97)], p = 0.0203, total of 799 events (79.4% of events in the mITT analysis), and the expanded coronary outcome [RR 0.85 (95% CI, 0.71–1.02)], p = 0.0723, total of 996 events (80.6% of events in mITT analysis) compared with the overall mITT analyses, as previously published [15].

**Fig. 2:**
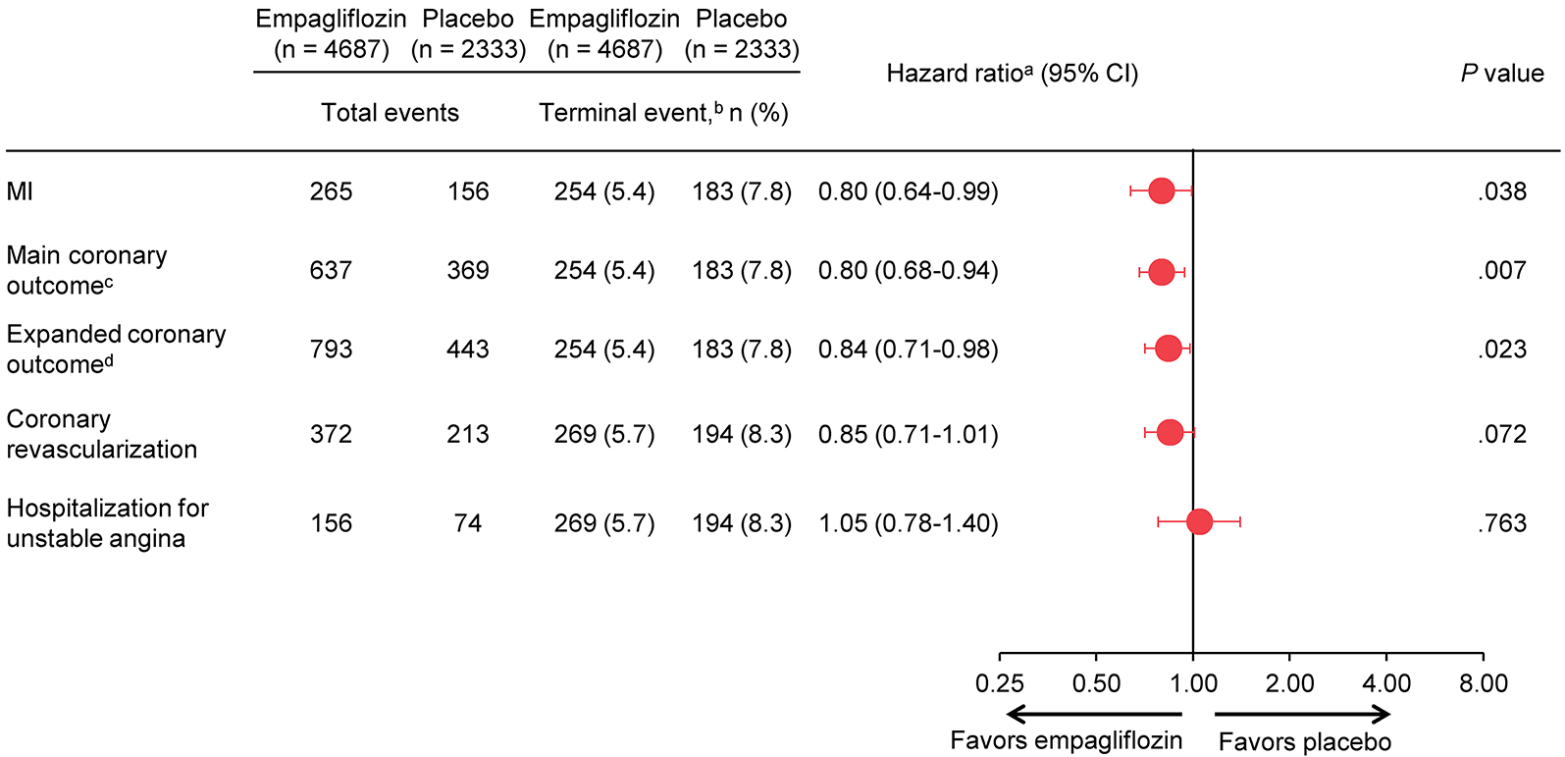
Risk of Total Coronary Events with Death/Death Other than Fatal MI as Terminal Event (Sensitivity Analysis). *CI* confidence interval, *MI* myocardial infarction. ^a^Joint frailty model includes treatment as a covariate. ^b^For MI, the main coronary outcome and the expanded coronary outcome, the terminal event was death other than fatal MI; for coronary revascularization and hospitalization for unstable angina, the terminal event was death. In the empagliflozin group, 269 patients (5.7%) died, with 254 (5.4%) dying from a cause other than fatal MI; in the placebo group, 194 patients (8.3%) died, with 183 (7.8%) dying from a cause other than fatal MI. ^c^MI or coronary revascularization. ^d^MI, coronary revascularization, or hospitalization for unstable angina

In sensitivity analyses using a joint frailty model, the treatment effects on the CV outcomes, including risk reductions for total MIs and the composite coronary outcomes, were consistent with the negative binomial model: hazard ratio for empagliflozin versus placebo of 0.80 (95% CI, 0.64–0.99) for MI, 0.80 (95% CI, 0.68–0.94) for the main coronary outcome, and 0.84 (95% CI, 0.71–0.98) for the expanded coronary outcome (Fig. 2).

## Discussion

Empagliflozin reduced the total burden of MIs and other coronary events by up to ~ 20% in T2D patients with atherosclerotic CV disease already receiving standard of care, including high use of other CV-protective therapies [15]. This effect was evident within ~ 3 months, was sustained, and was driven by the most common MI etiologies—those related to plaque-rupture/thrombus (type 1 MI) and supply–demand mismatch (type 2 MI). The treatment effect was consistent in subgroups according to baseline kidney function and various glucose-lowering therapies at baseline—including baseline use of GLP-1 analogues, and in on-treatment sensitivity analyses. Another SGLT inhibitor, dapagliflozin, demonstrated a significant reduction in the risk of first events of type 2 MIs in T2D patients with previous MI, with a directionally similar, but not statistically significant, effect on type 1 MIs [18]. While meta-analyses of SGLT inhibitors have shown mixed effects on first events of MIs [14, 19], total events analyses, like our study, support a reduced risk in T2D patients [6, 15]. A recent observational study suggested beneficial CV outcomes of the combination of SGLT inhibitors and GLP-1 analogue compared with either alone [20]. However, although we observed a consistent treatment effect of empagliflozin versus placebo in patients with baseline use of GLP-1 analogues in our analyses (all p for interaction > 0.05), the number of patients with baseline use of GLP-1 analogues was small [n = 196 (2.8%)] precluding conclusions.

Although the molecular mechanism of SGLT inhibitors to reduce glucose reabsorption in the kidneys is well established, the mechanism for their cardiorenal benefits remains unclear. However, SGLT inhibitors improve several metabolic and hemodynamic CV risk factors, including blood glucose, blood pressure, body weight, uric acid, oxidative stress, and inflammation [21], which may reduce atherosclerosis and thus risk of type 1 MI. Accordingly, recent studies have indicated that SGLT inhibitors may have anti-atherogenic effects, e.g., potentially less macrophage infiltration (inflammation) and lipid accumulation in the atherosclerotic plaque [22]. Likewise, SGLT inhibitors have been associated with lower risk of CV events, such as re-stenosis events, in T2D patients after coronary revascularization [23]. SGLT inhibitors also have effects that may increase supply of oxygen and nutrients to the heart—including increasing hemoglobin levels and shifting cardiac metabolism towards fatty acid and ketone substrates [21]. They also reduce plasma volume [24] and, consequently, may optimize left ventricular pre-load and after-load. Indeed, empagliflozin reduces the double product (heart rate × systolic blood pressure), a surrogate for myocardial oxygen demand [25]. Thus, SGLT inhibitors may improve cardiac supply–demand balance, with downstream effects in reducing type 2 MI. SGLT inhibitors have also been suggested to improve the myocardial microcirculation and consequently the supply of blood to the heart [26, 27]. Ultimately, such effects may have beneficial effects on both obstructive and non-obstructive coronary artery disease—in line with the consistent treatment effect of empagliflozin on type 1 and type 2 MIs observed in our analyses.

The strengths of our study include (1) pre-specification of total event analyses for MI, (2) centrally adjudicated outcomes, (3) statistical models preserving randomization and accounting for within-patient correlation of multiple events and different follow-up times, and (4) sensitivity analyses using alternative methods. Limitations include lack of (1) pre-specification for analysis of MI types and (2) adjustments for multiplicity. We also cannot exclude incomplete information on cause of death, e.g., sudden death. We did not have data on longer-term follow-up beyond the median of 3.1 years.

In conclusion, empagliflozin reduced total coronary events—including MI—in T2D patients with atherosclerotic CV disease, an effect that began early and was sustained. The reduction in MI was driven by reductions in both type 1 and 2 categories. Thus, the beneficial effects of empagliflozin may extend beyond reduced risk for CV mortality, heart failure and kidney disease to coronary outcomes in T2D patients with atherosclerotic CV disease.

### Supplementary Information

Supplementary file1 (DOCX 244 kb)

## Data Availability

To ensure independent interpretation of clinical study results and enable authors to fulfil their role and obligations under the ICMJE criteria, Boehringer Ingelheim grants all external authors access to relevant clinical study data. In adherence with the Boehringer Ingelheim Policy on Transparency and Publication of Clinical Study Data, scientific and medical researchers can request access to clinical study data after publication of the primary manuscript and secondary analyses in peer-reviewed journals and regulatory and reimbursement activities are completed, normally within 1 year after the marketing application has been granted by major regulatory authorities. Researchers should use the https://vivli.org/ link to request access to study data and visit https://www.mystudywindow.com/msw/datasharing for further information.

## Abbreviations

CI: Confidence interval
CV: Cardiovascular
MI: Myocardial infarction
RR: Relative risk
SGLT: Sodium-glucose transporter
T2D: Type 2 diabetes
