# Supplementary material for: Effect of empagliflozin on total myocardial infarction events by type and additional coronary outcomes: insights from the randomized EMPA-REG OUTCOME trial
Source: Cardiovasc Diabetol. 2024 Jul 11;23:248. doi: 10.1186/s12933-024-02328-6 (PMC11241944; doi:10.1186/s12933-024-02328-6)

**Supplemental Online Content**

**eFigure.** Mean Cumulative Function Curves for Total Number Of Events For A Myocardial Infarction, B Main Coronary Outcome, C Expanded Coronary Outcome, D Coronary Revascularization, E Hospitalization for Unstable Angina

**eFigure. Mean Cumulative Function Curves for Total Number Of Events For A Myocardial Infarction, B Main Coronary Outcome, C Expanded Coronary Outcome, D Coronary Revascularization, E Hospitalization for Unstable Angina**

We plotted the occurrence of total events over time using mean cumulative function plots that show the cumulative average number of events per patient. Rate ratios based on a negative binomial model including age as a linear covariate and treatment, sex, baseline BMI category, baseline HbA1c category, baseline eGFR category and geographical region as fixed effects with log (observation) time as offset. Abbreviations: BMI, body mass index; CI, confidence interval; eGFR, estimated glomerular filtration rate; HbA1c, glycated hemoglobin.

A Myocardial infarction


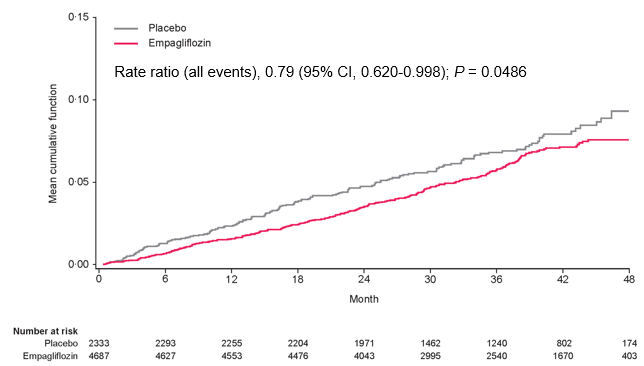


B Main coronary outcome (myocardial infarction and/or coronary revascularization)


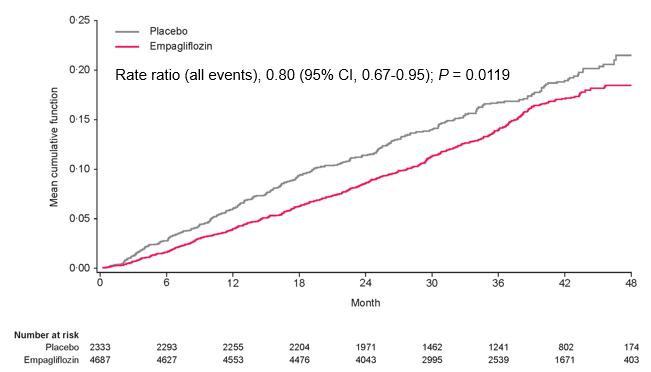


C Expanded coronary outcome (myocardial infarction and/or coronary revascularization and/or hospitalization for unstable angina)


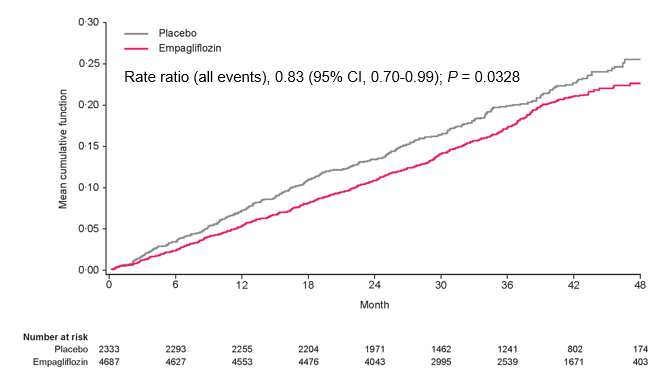


D Coronary revascularization


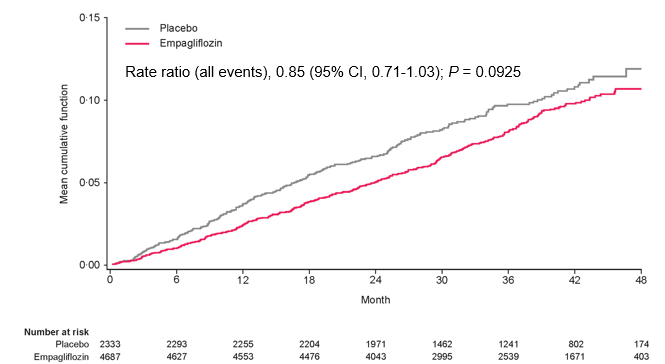


E Hospitalization for unstable angina


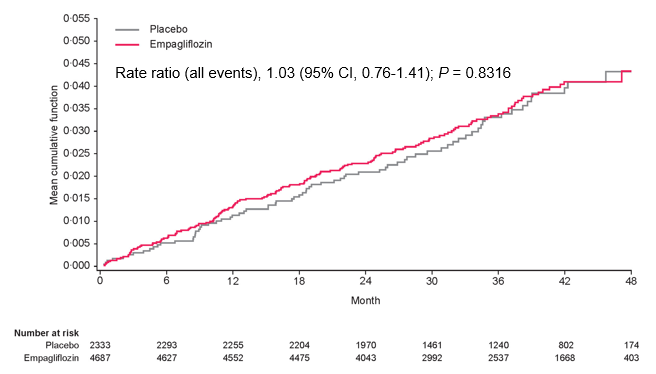

Supplement: Supplementary file 1 — Supplementary file1 (DOCX 244 kb) [file 12933_2024_2328_MOESM1_ESM.docx]
